# An Enhanced Approach Using AGS Network for Skin Cancer Classification

**DOI:** 10.3390/s25020394

**Published:** 2025-01-10

**Authors:** Hwanyoung Lee, Seeun Cho, Jiyoon Song, Hoyoung Kim, Youjin Shin

**Affiliations:** 1 Department of Computer Science and Information Engineering, The Catholic University of Korea, Bucheon 14662, Republic of Korea; aowlr1218@catholic.ac.kr; 2 Department of Artificial Intelligence, The Catholic University of Korea, Bucheon 14662, Republic of Korea; seeun1197@catholic.ac.kr (S.C.); sara4423@catholic.ac.kr (J.S.); 3 Department of Computer Science, Stony Brook University, Stony Brook, NY 11794, USA; hoyoung.kim.1@stonybrook.edu; 4 Department of Data Science, The Catholic University of Korea, Bucheon 14662, Republic of Korea

**Keywords:** skin cancer classification, medical image analysis, PGGAN, Unet

## Abstract

Skin cancer accounts for over 40% of all cancer diagnoses worldwide. However, accurately diagnosing skin cancer remains challenging for dermatologists, as multiple types of skin cancer often appear visually similar. The diagnostic accuracy of dermatologists ranges between 62% and 80%. Although AI models have shown promise in assisting with skin cancer classification in various studies, obtaining the large-scale medical image datasets required for AI model training is not straightforward. To address this limitation, this study proposes the AGS network, designed to overcome the challenges of small datasets and enhance the performance of skin cancer classifiers. The AGS network integrates three key modules: Augmentation (A), GAN (G), and Segmentation (S). It was evaluated using eight deep learning classifiers—GoogLeNet, DenseNet201, ResNet50, MobileNet V3, EfficientNet B0, ViT, EfficientNet V2, and Swin Transformers—on the HAM10000 dataset. Five model configurations were also tested to assess the contribution of each module. The results showed that all eight classifiers demonstrated consistent performance improvements with the AGS network. In particular, EfficientNet V2 + AGS achieved the most significant performance gains over the baseline model, with an increase of +0.1808 in Accuracy and +0.1674 in F1-Score. Among all configurations, ResNet50+AGS achieved the best overall performance, with an Accuracy of 95.87% and an F1-Score of 95.73%. While most previous studies focused on single augmentation methods, this study demonstrates the effectiveness of combining multiple augmentation techniques within an integrated framework. The AGS network demonstrates how integrating diverse methods can improve the performance of skin cancer classification models.

## 1. Introduction

Skin cancer is one of the most common types of cancer in the world. In the United States, more than 9500 people receive a skin cancer diagnosis each day, and over two lives are lost to the disease every hour [1,2,3]. The primary cause of skin cancer is ultraviolet (UV) radiation from sunlight, a proven human carcinogen that damages the immune system and induces mutations in tumor suppressor genes [4]. As this UV-induced damage accumulates over time, the likelihood of developing skin cancer increases with age. According to research [5], one in five Americans is expected to develop skin cancer by age 70. Therefore, accurate diagnosis of skin cancer is essential for improving public health in an aging population. In particular, early diagnosis is crucial because certain types, like melanoma, can spread (metastasize) to other organs, leading to potentially fatal outcomes. For example, the five-year survival rate of melanoma is over 99% when caught early, but this rate drops to 74% if the cancer has spread to the lymph nodes. Even if skin cancer is not life-threatening, early identification of the specific cancer type allows for patient-tailored treatments, which significantly improves the prognosis [6].

When symptoms of skin lesions appear, patients seek a diagnosis from a dermatologist. Dermatologists typically examine the skin using visual inspection or dermoscopy. Biopsies can also be performed, but they pose challenges in terms of time, complexity, and cost [7]. While visual or dermoscopy examinations are simple and cost-effective, they heavily rely on the dermatologist’s expertise and subjective interpretation. In addition, although dermoscopy enhances diagnostic accuracy compared to visual examination alone [8], sensitivity rates among dermatologists’ expertise often fail to exceed 80% [9]. Furthermore, a significant variance exists based on the level of training and professional experience [7].

To reduce diagnostic errors caused by subjectivity, AI-based diagnostic methodologies, including machine learning (ML) and deep learning (DL), are being developed to support dermatologists in making quicker and more reliable decisions [10,11]. These AI models are trained on vast datasets, far exceeding the number of cases a single expert might encounter in their career, thereby fulfilling the clinical need for diverse case exposure and enhancing diagnostic performance. For instance, one study demonstrated that with the assistance of an AI algorithm, the sensitivity and specificity of clinicians in malignancy prediction improved by 12.1% and 1.1%, respectively [12].

The application of machine learning (ML) and deep learning (DL) in medical diagnosis is becoming increasingly prevalent, yet achieving high-performance models remains a significant challenge. AI-based models are inherently data-driven, heavily reliant on the quality and quantity of available datasets. However, collecting large-scale image data for diverse skin cancers is particularly challenging due to the systemic constraints of medical data sharing. For instance, although the HAM10000 dataset took two decades to compile [13], its size remains insufficient for effectively training state-of-the-art AI algorithms. These challenges arise from privacy concerns, restrictive hospital policies, and the lack of standardization in data collection methods, which hinder the integration and sharing of medical datasets across institutions. Strict privacy laws, such as the General Data Protection Regulation (GDPR) and the Health Insurance Portability and Accountability Act (HIPAA), regulate how patient data can be shared, while hospital-specific restrictions and inconsistent data formats further complicate the process.

To address the limitations posed by the scarce availability of medical data, methodologies such as data augmentation, federated learning, and synthetic data generation are essential for maximizing data utility and supporting effective model development. Mohammed et al. achieved improved accuracy using a convolutional neural network (CNN)-based classifier with diverse datasets and appropriate balancing and augmentation strategies [14]. Additionally, Mikolajczyk, and Grochowski evaluated various data augmentation methods, including Generative Adversarial Networks (GANs), demonstrating the efficacy of newly generated images for pre-training neural networks, thus enhancing training efficiency [15]. However, these previous studies tend to focus on implementing a single approach in terms of data augmentation, with relatively few delving into the potential of integrated models that incorporate multiple techniques simultaneously to enhance their effectiveness. Most recent skin cancer studies [16,17,18,19] also utilize techniques such as segmentation and augmentation, but most adopt only one strategy. Hussain et al. [20] applied both lesion contrast enhancement and data augmentation; however, their work merely utilized basic implementations of these two techniques without exploring deeper integration. Furthermore, there has been limited analysis of their individual or collective contributions to performance improvements. This includes understanding how individual techniques influence overall performance and how their interactions collectively drive improvements.

In this study, we propose the AGS network, which integrates multiple modules to enhance data and improve prediction accuracy in the classification of skin lesions. Additionally, we analyze the individual and collective contributions of these modules to evaluate their impact on the overall model performance. The AGS network, the core of this classifier, consists of three modules: Module A (Augmentation), Module G (GAN), and Module S (Segmentation). Module A applies traditional image augmentation techniques such as flipping, rotation, and color jittering. Module G uses Progressive Growing of GAN (PGGAN) to generate high-quality synthetic images, mitigating the issue of limited dataset size. Modules A and G focus on generating diverse and abundant data, allowing AI models to be exposed to a wide range of cases. This capability might fulfill the clinical need for diverse case exposure. Module S employs the U-Net algorithm to segment the lesion area, removing background noise such as age spots and microscope artifacts, enabling more efficient feature learning. Similar to how dermatologists closely examine the lesion area for accurate diagnosis, Module S ensures that the AI model focuses on the critical parts of the lesion rather than the surrounding areas, enhancing its diagnostic precision. To evaluate the AGS network, we combined it with eight state-of-the-art deep learning models including GoogLeNet, DenseNet201, ResNet50, MobileNet V3, EfficientNet B0, ViT, Swin Transformer, and EfficientNet V2 and tested five different configurations: (1) the base model with raw data, (2) with Module A, (3) with Modules A and G, (4) with Modules A and S, and (5) the full AGS network with all three modules. The results showed that the AGS network performed best when all modules were used together. Finally, we employed Grad-CAM analysis to confirm that the AGS-enhanced classifier accurately focuses on lesion areas, reinforcing its clinical reliability. The contributions of our paper are summarized as follows:

- Introduction of the AGS Network: We suggest the AGS network, which combines three components—Augmentation (Module A), GAN (Module G), and Segmentation (Module S)—to improve skin lesion classification. These modules work together to increase data variability, ensure the model focuses on the critical lesion areas, and address dataset limitations. This network creates a more comprehensive dataset, significantly improving skin lesion classification.
- Performance Comparison Across Eight Classifiers: We evaluate the AGS network with eight well-known deep learning classifiers (GoogLeNet, DenseNet201, ResNet50, MobileNet V3, EfficientNet B0, ViT, Swin Transformer, and EfficientNet V2). The results showed that all eight classifiers demonstrated consistent performance improvements with the AGS network. ResNet50, when integrated with the AGS network, achieves the highest classification Accuracy, Precision, Recall, and F1-Score, making it the best-performing model.
- Comprehensive Analysis of Module Combinations: We conducted a detailed analysis of the impact of each module (A, G, and S) by testing five different configurations across eight classifiers. This ablation study revealed that the full AGS network (with all three modules) outperformed other configurations, highlighting the importance of combining all components.
- Grad-CAM Visualizations for Interpretability: Using Grad-CAM analysis, we validated that the AGS-enhanced classifier accurately identifies and focuses on the lesion areas in the images. This step adds an important layer of interpretability, making the AGS network more reliable for real-world clinical applications by ensuring that the model’s decisions are based on relevant medical features.

Through the proposed AGS network, it becomes possible to increase the amount of trainable data without violating the various policies governing sensitive medical data. This approach addresses the clinical need for diverse case exposure and contributes to enhancing classification performance. Furthermore, analyzing the individual and combined effectiveness of the AGS modules allows for a deeper understanding of the comprehensive impact on improving diagnostic accuracy. The organization of this paper is as follows: Section 2: This section reviews previous studies or literature related to the field of research and briefly compares the differences, advantages, and disadvantages between this study and previous ones. Section 3: This section describes the characteristics and limitations of the dataset used in the study, including the data preprocessing process. Section 4: This section provides detailed explanations of the method proposed in this study and the experimental environment setup. Section 5: This section presents the experimental results, verifying the performance of the proposed method. Section 6: In this section, the significance of the experimental results is discussed, along with an analysis of the strengths and limitations of the method proposed in this study. Section 7: This section provides a concise summary of the main findings and results of this study, and outlines how this research can contribute to the field of skin cancer classification using artificial intelligence.

## 2. Related Work

In recent years, many research studies have been conducted in the pursuit of diagnosing skin cancer. Some efforts to enhance diagnostic accuracy are presented in this section.

### 2.1. Skin Lesion Classification

Rahman et al. [21] proposed a weighted ensemble technique utilizing three deep learning pre-trained models—ResNet, Xception, and DenseNet—to classify skin lesion using the HAM10000 dataset, achieving a balanced accuracy of 85.8%. Emrah et al. [22] employed the pre-trained VGGNET-16 architecture using with the HAM10000 dataset. To partition the dataset for training and testing, the K-Fold Cross-Validation method was adopted and the model achieved an 85.62% accuracy rate. Chaturvedi, Gupta, and Prasad [23] employed transfer learning, utilizing a MobileNet model pre-trained on 1.28 million images from the 2014 ImageNet Challenge and fine-tuned the model using 10015 dermoscopy images from the HAM10000 dataset. This model obtained an overall accuracy of 83.1% for seven classes. In 2017, Esteva et al. [24] achieved a significant advancement in skin lesion classification using the Inception v3 architecture, training on macroscopic and dermoscopic images. The author mapped 2032 diseases into 757 classes, achieving superior results. In 2020, Syed, Shyla, and Mehera [25] used fine-tuned DenseNet-121 to classify seven types of classes with the HAM10000 dataset. In medical image analysis [26], pre-trained deep CNNs, when adequately fine-tuned, have shown to either outperform or match the performance of CNNs trained from scratch across various applications, demonstrating greater robustness to training set sizes and highlighting the effectiveness of a tailored layer-wise fine-tuning approach. Olusola et al. [27] introduced a effective approach to skin cancer detection, particularly melanoma, by employing a data augmentation technique based on SMOTE, utilizing the publicly available $PH^{2}$ dataset for training. The methodology demonstrated significant improvements in accuracy, sensitivity, and F1-score for melanoma detection, showcasing enhanced performance in multi-class classification. This paper [28] suggested advancements in data augmentation techniques for MRI brain tumor images to understand their impact on supervised learning models. This study [29] systematically examined the relationship between ImageNet performance and transfer learning across 16 classification networks and 12 image classification dataset, finding a strong correlation between ImageNet accuracy and transfer accuracy. Anand et al. [30] presented a deep learning model using transfer learning for the early detection of skin cancer, accomplishing an overall accuracy of 89.08%. The model, based on a pre-trained VGG16 architecture enhanced with additional layers and LeakyReLU activation functions, outperformed existing methods.

Anand, Gupta, and Koundal [31] suggested a modified MobileNet architecture for skin disease classification, removing the last five layers and incorporating an average pooling layer, dropout layer, and dense layer. Utilizing the HAM10000 dataset and various transformation techniques for data augmentation, the model gained a 90% accuracy and an 86.14% precision, addressing issues of data imbalance. Mohammed et al. [14] introduced an integrated diagnostic framework for skin lesion analysis, combining boundary segmentation and lesion classification utilizing various convolutional neural networks. Through segmentation with FrCN and classification with CNN classifiers, the model achieved improved accuracy across ISIC 2016, 2017, and 2018 datasets with proper balancing and augmentation techniques, demonstrating the effectiveness of the proposed approach in enhancing lesion diagnosis accuracy. In 2021, Khan et al. [32] proposed an automated approach for skin lesion classification, accomplishing a classification accuracy of 90.67% on the HAM10000 dataset. Salian, Vaze, and Singh [33] achieved over 80% accuracy in classifying skin lesion types with the $PH^{2}$ dataset and HAM10000 dataset, using MobileNet, VGG-16, and a custom model. Khan, Zhang, and Sharif et al. [16] presented a deep learning framework integrating MASK R-CNN for lesion segmentation and a 24-layered CNN for classification. Evaluation on the HAM10000 dataset for classification demonstrated an 86.5% accuracy.

Ayas et al. [17] proposed a Swin Transformer model for multi-class skin lesion classification, leveraging Transformers and CNNs to address inter-class similarity and intra-class variation. This method was validated on the ISIC 2019 dataset and effectively managed class imbalance using weighted cross-entropy loss. Gangwani et al. [34] employed a generic CNN and demonstrated that transfer learning with a pre-trained framework enhances detection accuracy. Furthermore, data augmentation techniques were employed to improve model performance. Through experimentation with different parameters, including learning rate, batch sizes, and optimizers, they reached 91% accuracy on the HAM10000 dataset. Yusuf et al. [18] utilized EfficientNetV2-B2 for skin lesion classification on the HAM10000 dataset, employing extensive hyperparameter tuning and image augmentation. The model demonstrated robust performance with an Accuracy of 89%, Precision of 81%, Recall of 80%, and F1-Score of 80%. Hussain et al. [20] proposed the SkinNet-INIO model, which integrates Fusion-Assisted Deep Neural Networks with an Improved Nature-Inspired Optimization (INIO) algorithm for multi-class classification and localization of skin lesions. The framework enhances contrast using a dark channel haze and top–bottom filtering technique, and features are fused through a serial correlation-based approach. The model was validated on the ISIC2018 and ISIC2019 datasets, showing improved performance over state-of-the-art methods. Khan et al. [19] introduced MSRNet, a deep learning architecture for multi-class skin lesion classification and melanoma detection. The model employs pre-trained DarkNet-53 and DenseNet-201 networks fine-tuned with transfer learning and optimized using a Genetic Algorithm (GA). A two-step serial–harmonic mean approach is used for feature fusion, while Marine Predator Optimization (MPA) refines feature selection. The model achieved competitive results on the ISIC2018 and ISIC2019 datasets. To aid understanding, relevant studies on skin lesion prediction using the HAM10000 dataset, as covered in this study, are summarized in Table 1.

### 2.2. Data Augmentation and Segmentation

Mohamed et al. [35] utilized MobileNet and DenseNet-121 to compare the accuracy differences when training on a downsampled balanced dataset versus an imbalanced original dataset. As a result, they achieved 82.6% and 71.9% accuracy, respectively, on the test image set, while on the original dataset, the accuracy was 92.7% and 91.2%. Mikolajczyk and Grochowski [15] considered various data augmentation methods for image classification, ranging from traditional transformation to advanced techniques like Style Transfer and Generative Adversarial Networks (GANs), and introduced a novel approach based on image style transfer, enhancing perceptual quality by combining content from one image with the appearance of others. This method was validated across three medical case studies, addressing data scarcity in medical image analysis. Wannipa et al. [36] introduced a modified MobileNet architecture for skin lesion classification, enhancing the conventional MobileNet by adjusting its layers and reducing the parameter count for improved efficiency. Additionally, the effect of data upsampling and augmentation techniques on both MobileNet and the suggested version were investigated. Aminet al. [37] conducted a comparison of six well-known sampling techniques designed to address class imbalance issues, including the mega-trend diffusion function (MTDF), synthetic minority oversampling technique (SMOTE), and four others. In this work [38], AUGMIX, an efficient data processing technique, was introduced to enhance the robustness and uncertainty estimates of image classifiers. It demonstrates significant improvements on image classification, in some instances reducing the performance gap with the best achievable outcomes. Zhong et al. [39] presented Random Erasing, a novel data augmentation technique for CNN training that randomly erases parts of an image to generate various levels of occlusion. Despite its simplicity, Random Erasing effectively complements existing augmentation methods like random cropping and flipping, consistently improving performance in image classification, object detection, and person re-identification. Wang et al. [40] investigated various data augmentation techniques for image classification, focusing on a constrained subset of the ImageNet dataset. Traditional transformations like cropping, rotating, and flipping were compared alongside experiments with GAN for generating images. Additionally, neural augmentation was introduced, a novel method enabling a neural network to learn the most effective augmentations for improving classifier performance. Alptekin et al. [41] investigated the impact of data augmentation on deep learning models for skin lesion classification, observing positive contributions to model performance. Alam et al. [42] employed data augmentation to address the class imbalance problem with the HAM10000 dataset. Furthermore, they proposed a framework that achieved an accuracy of 91%. Alomar et al. [43] proposed a technique utilizing local image information for augmentation, presenting a random local rotation strategy.

Anand et al. [44] introduced a modified U-Net architecture for precise and automatic segmentation of dermoscopic images by adjusting the feature map’s dimensions. Sumithra et al. [45] employed a region growing approach for segmentation of skin lesions by automatically initializing seed points and used two classifiers for classification. Javaid, Sadiq, and Akram [46] proposed a method for skin lesion segmentation using the OTSU thresholding algorithm. In 2018, Vesal et al. [47] presented a modified version of U-Net incorporating dilated and dense block convolutions to obtain multi-scale and global context information. Chen et al. [48] proposed DeepLab, a state-of-the-art semantic segmentation framework designed to address the challenges of pixel-level image classification. By integrating atrous convolution to capture contextual information at multiple scales and employing fully connected Conditional Random Fields (CRFs) to refine segmentation boundaries, DeepLab achieved significant advancements in accuracy and precision. This framework has been extensively validated and is widely recognized for its effectiveness in medical image analysis, particularly in accurately segmenting complex and heterogeneous regions. He et al. [49] introduced Mask R-CNN, an advanced extension of Faster R-CNN tailored for object instance segmentation. This architecture integrates an additional mask branch that operates concurrently with bounding box regression and class prediction, enabling the precise generation of segmentation masks for each detected object. Mask R-CNN has been widely adopted in dermoscopic image analysis, where it excels at delineating lesion boundaries with high accuracy, thereby significantly improving lesion classification workflows.

## 3. Dataset

**Figure 1:**
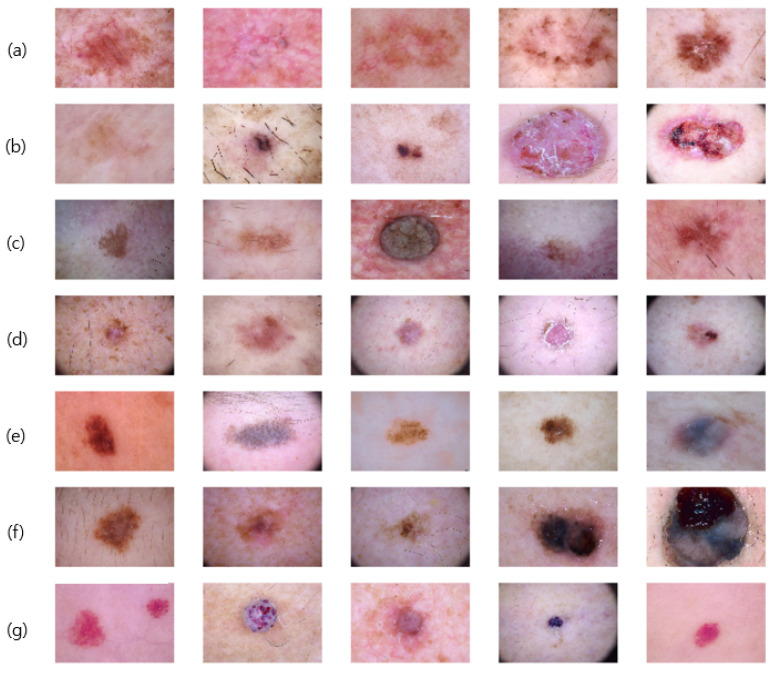
HAM 10000 dataset classified into seven categories: (**a**) Actinic Keratosis (AKIEC), (**b**) Basal Cell Carcinoma (BCC), (**c**) Benign Keratosis (BKL), (**d**) Dermatofibroma (DF), (**e**) Melanocytic Nevi (NV), (**f**) Melanoma (MEL), and (**g**) Vascular Lesions (VASCs).

To develop an AI-based algorithm for skin cancer prediction, we use the Human Against Machine with 10,000 training images (HAM10000) dataset [13], which is a benchmark dataset for skin lesion classification, encompassing images sourced from patients in Australia and Austria. The dataset comprises 10,015 skin lesion images, categorized into seven types of skin lesions as illustrated in Figure 1. The benign categories include melanocytic nevi (NV, 6705 images), actinic keratosis and intraepithelial carcinoma (AKIEC, 327 images), dermal fibroma (DF, 115 images), vascular lesions (VASC, 142 images), and benign keratosis-like lesions (BKL, 1099 images). Conversely, the malignant categories consist of basal cell carcinoma (BCC, 514 images) and melanoma (MEL, 1113 images).

The acquisition of substantial data for training deep learning models remains a formidable challenge, particularly within the intricate domain of medical imaging. The inherent difficulty in procuring medical data accentuates the scarcity issue, hindering the development of robust classification models.

To address the inherent data scarcity in training robust skin cancer classification models, we developed an AGS network consisting of three modules: Augmentation (Module A), GAN (Module G), and Segmentation (Module S). Consequently, augmenting datasets assumes a pivotal role in mitigating the risk of overfitting and increasing the generalization capabilities of classification models. Detailed methodologies for our AGS network are explained in Section 4.

## 4. Methods and Experiments

In this section, we provide a detailed description of our novel skin cancer classification model incorporating the AGS network. First, in Section 4.1, *Overall Architecture*, we introduce the overall design and algorithm of our model. Next, in Section 4.2, *AGS Network*, we delve into the A, G, and S modules that constitute the core of our model. In Section 4.3, *Classifier Selection*, we discuss the selection of the classifier model from eight deep learning algorithms. Finally, in Section 4.4, *Experiments*, we describe the experiments conducted to demonstrate the effectiveness of the AGS network and the overall classification performance.

### 4.1. Overall Architecture

**Figure 2:**
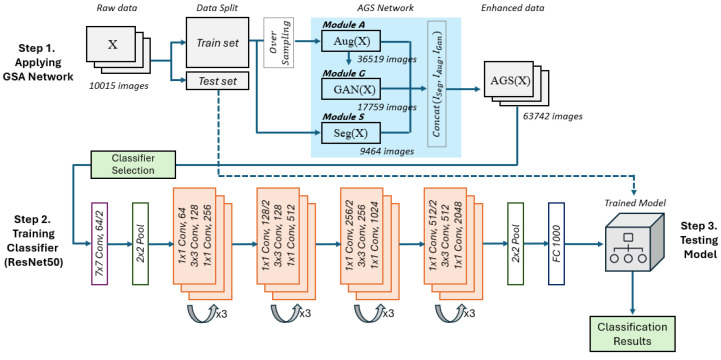
The overall architecture of the proposed model with the AGS network for skin lesion classification.

Our skin cancer classification model with the AGS network consists of three major steps, as shown in Figure 2. Step 1 begins with dataset preparation, where 10,015 skin cancer images are collected, normalized by scaling pixel values to have a mean of 0 and a standard deviation of 1, and then divided into a training set and a test set. The training set is processed through the AGS network after oversampling, which is the core component of our methodology, designed to enhance the data for more effective learning. The test set is kept separate for final model evaluation, ensuring that it is never utilized during the training phase. The AGS network marked with a blue box consists of three distinct modules. First, Module A performs basic data augmentation, including horizontal flipping, vertical flipping, random rotation, and color jittering, to diversify the training data. Specifically, horizontal and vertical flips are applied randomly, random rotation is performed within an angle range of −20 to 20 degrees, and color jitter adjusts the brightness, contrast, and hue by up to 10%. In total, 36,519 images are processed through this module, with flip, rotation, and jittering applied randomly. Second, Module G employs a generative network, specifically PGGAN (Progressive Growing of GANs), to generate 17,759 synthetic yet realistic samples. PGGAN progressively increases the resolution of the generated images, ensuring high-quality and detailed synthetic samples that closely resemble real skin cancer lesions, thus further enriching the dataset. Lastly, Module S focuses on extracting critical features by identifying and highlighting the core regions of cancerous lesions, utilizing 9464 images in this segmentation process. The core region in the segmentation process is defined based on the initial binary mask generated from the network output. This binary mask is created by applying a threshold to the pixel-wise probabilities produced by the network. The largest continuous region is then considered the core region. After processing through each module, the outputs are combined into a single enhanced dataset through a concatenation layer, ensuring the model is exposed to a diverse and comprehensive training set. Detailed descriptions of Module A, Module G, and Module S are provided in Section 4.2.

In Step 2 in Figure 2, the enhanced dataset produced by the AGS network is used to train a classifier. At this stage, we experiment with eight leading deep learning algorithms known for their effectiveness in image classification tasks. This empirical approach allows us to identify the algorithm that best complements the AGS-enhanced data, which is then selected as the final classifier. Step 3 in Figure 2 involves evaluating the performance of the trained model using the test set. The evaluation is conducted by measuring four key metrics: Accuracy, Precision, Recall, and F1-Score. These metrics are calculated using the Mean Squared Error (MSE) to ensure a robust assessment of the model’s performance. To guarantee a fair and unbiased evaluation, the test set was split while maintaining the original data distribution before training, and then we employ 5-fold stratified cross-validation, testing the model on five different test sets and reporting the average performance. Further details of the evaluation process are provided in Section 5.

### 4.2. AGS Network

Our AGS network is a novel approach designed to enhance the classification accuracy of models by incorporating diverse data augmentation techniques. It consists of three components: Module A for traditional data augmentation, Module G for generating images, and Module S for segmenting important regions. Each of the three modules will be introduced in detail below. Further details on the implementation of our AGS network, including code and additional resources, can be found in our GitHub repository [50].

**Module A (Augmentation)**: Traditional data augmentation techniques, such as random horizontal flipping, random vertical flipping, random rotations, and color jittering, are applied to the original images. These techniques enhance the classifier’s ability to generalize by exposing it to varied orientations, perspectives, and lighting conditions. This increased exposure helps in building robust classifiers that perform well across diverse inputs, thereby reducing overfitting and improving overall performance metrics on new data. Moreover, data augmentation optimizes data efficiency by expanding the effective dataset size without requiring additional data collection efforts. We define the input images as $I_{RGB}$, which can be expressed as Equation (1), where $a_{i,j}$ denotes the pixel value at position (i,j)∈Ω, with $\Omega \in Z^{2}$ representing the set of all pixel positions in the image. Details of the four different augmentation techniques we applied are provided below.

(1) $$I_{RGB}=\left\{\begin{array}{cccccc} a_{0,0} & . & . & . & a_{0,j} & \\ . & . & . & . & . & \\ . & . & . & . & . & \\ . & . & . & . & . & \\ a_{i-1,0} & . & . & . & a_{i-1,j} & \\ a_{i,0} & . & . & . & a_{i,j} & \end{array}\right\}$$

- Random Horizontal Flipping: We randomly flip an image along its vertical axis, creating a mirror image. This transformation reflects the pixel at (i,j) across the y-axis. As a result, the horizontal flip (*HF*) results in the image being flipped left-to-right with a given probability *p* as shown in Equation (2).
  (2) $$HF\left(I_{RGB},p\right)=\left\{\begin{array}{cccccc} a_{0,j} & . & . & . & a_{0,0} & \\ . & . & . & . & . & \\ . & . & . & . & . & \\ . & . & . & . & . & \\ a_{i-1,j} & . & . & . & a_{i-1,0} & \\ a_{i,j} & . & . & . & a_{i,0} & \end{array}\right\}$$
- Random Vertical Flipping: We apply a random vertical flip (*VF*), a transformation that reflects the value (i,j) across the x-axis. This operation flips the image upside down with a given probability *p*. The vertically flipped image can be represented as shown in Equation (3).
  (3) $$VF\left(I_{RGB},p\right)=\left\{\begin{array}{cccccc} a_{i,0} & . & . & . & a_{i,j} & \\ a_{i-1,0} & . & . & . & a_{i-1,j}\\ . & . & . & . & . & \\ . & . & . & . & . & \\ . & . & . & . & . & \\ a_{0,0} & . & . & . & a_{0,j} & \end{array}\right\}$$
- Random Rotation: The rotation matrix for performing a rotation transformation by an angle θ can be expressed as Equation (4). In this case, θ is assumed to be positive, which corresponds to a counterclockwise rotation. By multiplying the pixel value (*i*, *j*) of the input image by rotation matrix, the value ($\hat{i},\hat{j}$) of the rotated image can be obtained. This value is represented by Equation (5).
  (4) $$R\left(\theta \right)=\left(\begin{array}{ccc} \cos\theta & -\sin\theta & 0\\ \sin\theta & \cos\theta & 0\\ 0 & 0 & 1 \end{array}\right)$$
  (5) $$\begin{array}{c} \hat{i}=i\cos\theta +j\sin\theta \\ \hat{j}=-i\sin\theta +j\cos\theta \end{array}$$
- Color Jittering: We apply changes in brightness $\delta_{b}$, contrast $\delta_{c}$, saturation $\delta_{s}$ and hue $\delta_{h}$ to the pixel values of the input image $I_{RGB}$. This technique introduces variability into the dataset, which enriches its diversity. Brightness adjustment modifies luminance, helping the model recognize objects in varying lighting. Contrast variation increases the disparity between light and dark areas, emphasizing features. Saturation modification adjusts the richness of colors, allowing the model to remain effective across different levels of color intensity, while hue transformation shifts the color spectrum, reducing sensitivity to color biases. By integrating these transformations, color jittering improves model performance and robustness.
  $I_{brightness}$ is calculated as follows when the image $I_{RGB}$ and brightness $\delta_{b}$ are given as Equation (6). This formula increases the brightness of the image by a value of $\delta_{b}$, resulting in each pixel value being multiplied by $\delta_{b}$. The resulting values are then clamped between 0 and the maximum pixel value of $I_{RGB}$.
  (6) $$I_{brightness}=\min\left(\max\left(\delta_{b}\;\cdot \;I_{RGB},0\right),\max\left(I_{RGB}\right)\right)$$
  Equation (7) adjusts the color saturation of an image, making the colors appear stronger or weaker. If $\delta_{s}$ is 0, the image is converted to grayscale, while a value of 1 retains the original image. This function is used to visually alter the intensity of colors. The resulting values are then clamped between 0 and the maximum pixel value of $I_{RGB}$.
  (7) $$\begin{array}{c} I_{saturation}=\min\left(\max\left(\delta_{s}\;\cdot \;I_{RGB}+\left(1-\delta_{s}\right)\;\cdot \;I_{gray},0\right),\max\left(I_{RGB}\right)\right),\\ where\;I_{gray}=\left(0.2989\;\cdot \;I_{R}+0.587\;\cdot \;I_{G}+0.114\;\cdot \;I_{B}\right) \end{array}$$
  Equation (8) is used to adjust the contrast of an image. It modifies the image by blending the original image with its mean brightness, either lowering or increasing the contrast. When the $\delta_{c}$ is 1, the original image is preserved. A value of 0 transforms the image entirely to its mean brightness. Values greater than 1 increase contrast, while values between 0 and 1 decrease it. This allows for adjustment of the visual characteristics of the image. As contrast increases, dark areas become darker and bright areas become brighter. When contrast decreases, the difference between light and dark areas is reduced, resulting in a flatter image. And the resulting values are then clamped between 0 and the maximum pixel value of $I_{RGB}$.
  (8) $$\begin{array}{c} I_{contrast}=\min\left(\max\left(\delta_{c}\;\cdot \;I_{RGB}+\left(1-\delta_{c}\right)\;\cdot \;I_{mean},0\right),\max\left(I_{RGB}\right)\right),\\ where\;I_{mean}=\frac{1}{i\;\cdot \;j}\sum_{x=0}^{i}\sum_{y=0}^{j}I_{gray}\left(x,y\right) \end{array}$$
  For calculating $I_{hue}$, we perform the process of converting the input RGB image into the HSV color space, followed by adjusting the H (hue) value. The individual color channels of the RGB image are analyzed, facilitating the transformation into the HSV color space. The H value is then modified according to $\delta_{h}$ as shown in Equation (9). Subsequently, using the $H_{adjusted}$, the image is transformed back into the RGB color space to produce $I_{hue}$.
  (9) $$H_{adjusted}=\mathrm{mod}\left(H+\delta_{h},1\right)$$
  Consequently, the image modified through ColorJitter can be expressed as Equation (10).
  (10) $$ColorJitter\left(I_{RGB},\delta_{b},\delta_{c},\delta_{h},\delta_{s}\right)=\left\{\begin{array}{c} I_{brightness}\\ I_{saturation}\\ I_{contrast}\\ I_{hue} \end{array}\right\}$$

In conclusion, the final result of applying augmentation techniques such as Random Horizontal Flipping, Random Vertical Flipping, Random Rotation, and Color Jittering to the $I_{RGB}$ is represented by $I_{A}$ in Module A.

**Figure 3:**
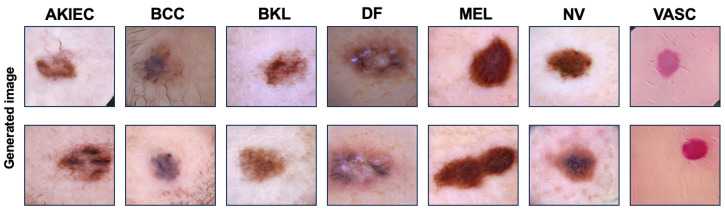
HAM10000 dataset classified into 7 classes and images generated using PGGAN.

**Module G (GAN)**: We employ Progressive Growing of GANs (PGGAN) [51] to generate images resembling the features present in the HAM10000 dataset, as illustrated in Figure 3. PGGAN is an evolved version of the original Generative Adversarial Network (GAN), which consists of a Generator and a Discriminator engaged in a minimax game. While GANs can effectively generate data by learning the underlying distribution of a dataset, they often face issues such as training instability and difficulty in generating high-resolution images. PGGAN addresses these limitations by introducing a progressive training approach. It begins by generating low-resolution images and gradually increases the resolution as training progresses, allowing for more stable convergence and better performance with high-resolution outputs. Additionally, PGGAN adopts the Wasserstein GAN with Gradient Penalty (WGAN-GP) as its loss function as shown in Equation (11). This improves training stability by minimizing the Wasserstein distance between the real and generated data distributions and applying a gradient penalty to ensure smoother gradients.

(11) $$\min_{G}\max_{D}E_{x∼p_{data}}\left[D\left(x\right)\right]-E_{z∼p_{z}}\left[D\left(G\left(z\right)\right)\right]+\lambda E_{\hat{x}∼p_{\hat{x}}}\left({\left(∥\nabla_{\hat{x}}D\left(\hat{x}\right){∥}_{2}-1\right)}^{2}\right),$$

where $E_{x∼p_{data}}\left[D\left(x\right)\right]$ is the expectation of the critic’s output for real data samples *x* drawn from the real data distribution $p_{data}$, and $E_{z∼p_{z}}\left[D\left(G\left(z\right)\right)\right]$ represents the expectation of the critic’s output for generated data G(z) from noise *z* sampled from the noise distribution $p_{z}$. The third term $\lambda E_{\hat{x}∼p_{\hat{x}}}{\left(∥\nabla_{\hat{x}}D\left(\hat{x}\right){∥}_{2}-1\right)}^{2}$ is the gradient penalty, where $\hat{x}$ is interpolated between real and generated data samples, $\nabla_{\hat{x}}D\left(\hat{x}\right)$ is the gradient of the critic with respect to $\hat{x}$, and λ is a regularization parameter that controls the strength of the penalty.

As input data to PGGAN, we used transformed images through Module A, rather than raw data, in order to maintain image diversity. The final augmented image through PGGAN is denoted as $I_{G}$ in Module G.

**Figure 4:**
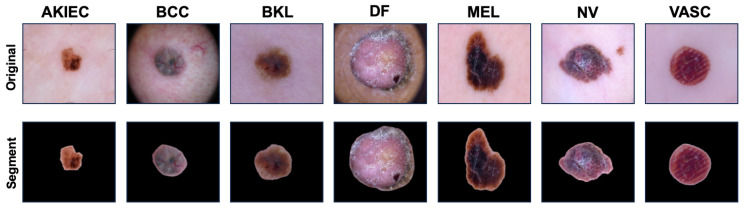
HAM10000 dataset classified into 7 classes and images segmented using U-Net.

**Module S (Segmentation)**: The HAM10000 dataset consists of images focused on lesions, but in some cases, a significant portion of the image is occupied by background regions that do not contain important information. Therefore, we isolate and extract regions of interest, such as lesions, from the original images as illustrated in Figure 4. This segmentation process is crucial as it directs the model’s attention to pertinent features essential for accurate classification, thereby enhancing its robustness against irrelevant background noise. To determine the most suitable segmentation model, we compared the performance of DeepLab [48], Mask R-CNN [49], and U-Net [52] using Intersection-over-Union (IoU) and the Dice coefficient as evaluation metrics. Among these models, U-Net achieved the highest performance on both metrics as shown in Table 2, leading us to adopt it as our segmentation algorithm.

The area of the lesion to be segmented was defined with the segmentation network as described by Tschandl et al. [53]. The core region in the segmentation process is defined based on the initial binary mask generated from the network output. This binary mask is created by applying a threshold to the pixel-wise probabilities produced by the network. The largest continuous region is then considered the core region. U-Net follows an encoder–decoder structure, where the encoder progressively downsamples the input image through a series of convolutional and pooling layers, while the decoder upsamples the feature maps to reconstruct the spatial dimensions. One key element of U-Net is the incorporation of skip connections, which directly transfer feature maps from the encoder to the corresponding decoder layers, ensuring the preservation of fine-grained spatial information. Mathematically, the output at the *i*-th layer of the encoder is computed as follows:

(12) $$f_{i}=\sigma \left(W_{i}*f_{i-1}+b_{i}\right),$$

where $f_{i}$ represents the feature map at layer *i*, $W_{i}$ is the convolutional filter, $b_{i}$ is the bias term, and σ is the non-linear activation function (e.g., ReLU). The * symbol denotes the convolution operation.

In the decoder, the corresponding upsampling operation is expressed as follows:

(13) $$f_{i}^{\prime }=\sigma \left(W_{i}^{\prime }*f_{i+1}^{\prime }+b_{i}^{\prime }\right),$$

where $f_{i}^{\prime }$ is the feature map at layer *i* in the decoding path, and $W_{i}^{\prime }$ is the transposed convolution filter used to upsample the feature map.

The skip connection, which merges encoder and decoder feature maps, is defined as follows:

(14) $$f_{i}^{\prime \prime }=\mathrm{Concat}\left(f_{i},f_{i}^{\prime }\right),$$

where $f_{i}^{\prime \prime }$ is the concatenated feature map at layer *i*, combining the encoder’s feature map $f_{i}$ with the decoder’s upsampled feature map $f_{i}^{\prime }$. This skip connection allows the network to leverage both high-level semantic information from the decoder and fine-grained details from the encoder, leading to more accurate segmentation results.

Finally, for pixel-wise classification, a softmax activation function is applied to the final output layer to generate probabilities for each class. The softmax function is defined as follows:

(15) $$p_{k}\left(x\right)=\frac{\exp(a_{k}\left(x\right))}{\sum_{k^{\prime }=1}^{K}\exp\left(a_{k^{\prime }}\left(x\right)\right)},$$

where $p_{k}\left(x\right)$ represents the probability that pixel *x* belongs to class *k*, $a_{k}\left(x\right)$ denotes the activation value for class *k* at pixel *x*, and *K* is the total number of classes. This ensures that the sum of probabilities for all classes at each pixel equals 1, and the class with the highest probability is selected as the final prediction.

Such structural characteristics make U-Net particularly suitable for dermatological image analysis, enabling precise learning of intricate lesion boundaries and shapes. By using U-Net, our Module S ensures accurate delineation of lesions, thereby empowering the classifier to extract and utilize critical features essential for precise classification of various skin lesions. The final segmented image via U-Net is denoted as $I_{S}$ in Module S.

Finally, as $Image_{A}$, $Image_{G}$, and $Image_{S}$ represent the outputs from the Module A, Module G, and Module S, respectively, our AGS network can be expressed through concatenation as follows:

(16) $$Image_{AGS}=Concat\left(Image_{A},Image_{G},Image_{S}\right)$$

### 4.3. Classifier Selection

After generating the enhanced data through the AGS network, we conducted experiments using eight different deep learning algorithms suitable for skin lesion image classification to train the classifier, as shown in Step 2 in Figure 2. Although one model with the best performance among the eight is selected as the final classifier, all eight models are also used in experiments to demonstrate the utility of the AGS network. Further details will be explained in Section 4.4. The eight deep learning models we used in our experiments are as follows:

- GoogLeNet [54]: it employs inception modules, utilizing kernels of various sizes simultaneously to effectively extract features of different scales.
- DenseNet201 [55]: it maintains rich features in the network by connecting each layer’s input to the output of the current layer, thus preserving features from previous layers while adding new ones.
- ResNet50 [56]: it alleviates the gradient vanishing problem effectively by employing skip connections, enabling effective training of deep networks.
- MobileNetV3 [57]: it minimizes model size and computational load using depthwise separable convolutions.
- EfficientNetB0 [58]: it enhances computational efficiency through automated model scaling methods and specialized operators.
- ViT [59]: it leverages a patch-based approach combined with the transformer architecture to capture global information within images.
- Swin Transformer [60]: it adopts a hierarchical structure using shifted windows, enabling efficient modeling of long-range dependencies while preserving computational efficiency for large-scale vision tasks.
- EfficientNetV2 [61]: it incorporates advanced training optimization techniques and a refined architecture to improve computational efficiency and training speed for image classification tasks.

There are different versions of the algorithms with slight variations in the layer structure or the number of layers. We experimented with multiple configurations and selected the one with the best performance. For example, in the case of ResNet, we tested ResNet18, ResNet34, ResNet50, ResNet101, and ResNet152, and ultimately chose ResNet50, which demonstrated the best performance.

### 4.4. Experiments

We conducted an experiment to compare the performance of the eight models mentioned in Section 4.3, with and without the AGS network. For the dataset, we used the HAM10000, which comprises 10,015 skin lesion images categorized into 7 different types, as described in Section 3. In this study, around 35,000 images were generated through Module G and 10,000 images through Module S considering the ratio between classes.

Given the relatively small size of the dataset, we employed 5-fold cross-validation to ensure robust performance evaluation. In this process, the data are split into five subsets, and the model is trained and tested five times, each time using a different subset for testing while the remaining four are used for training and validation. This allows the model to be tested on all data points, and the final performance is reported as the average of the five runs. Specifically, we used 80% of the data for training, 10% for validation, and 10% for testing in each fold. The models were trained using the Adam optimizer and cross-entropy loss function. More details are shown in Table 3.

**Figure 5:**
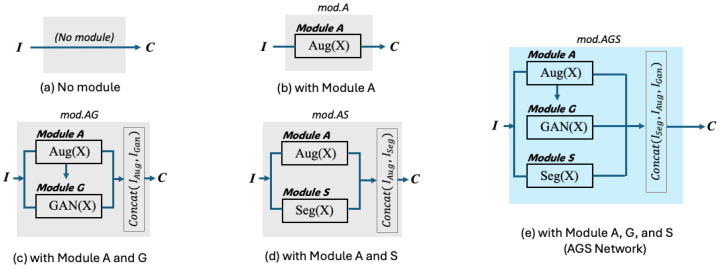
Architecture of various module combinations. To determine the effectiveness of module combinations, five configurations were designed and their performance was compared. “I” denotes the input data, while “C” represents the classifier. The performance for each combination from (**a**–**e**) is described in order in Table 6. Classifiers C∈{GoogleNet,DenseNet201,ResNet50,MobileNetv3,EfficientNetb0,ViT,SwinTransformer,EfficientNetV2} were used for evaluation.

An ablation study was also conducted to analyze the impact of each module in the AGS network on performance improvement. We conducted experiments with various combinations of modules to evaluate the performance of the AGS network in improving skin lesion classification. The experiments involved five different configurations: (1) the base model, (2) the model with Module A, (3) the model with both Modules A and G, (4) the model with both Modules A and S, and (5) the model with Modules A, G, and S, which is our proposed AGS network. The detailed architecture for the five different configurations is illustrated in Figure 5.

For each of the five module configurations, we tested eight deep learning algorithms as described in Section 4.3: GoogLeNet, DenseNet201, ResNet50, MobileNetV3, EfficientNetB0, ViT, Swin Transformer, and EfficientNetV2. By evaluating all combinations of module configurations and classifiers, we aimed to identify the optimal setup for improving skin lesion classification.

Additionally, to verify whether our model focuses on the core areas of each lesion, we conducted experiments generating heatmaps using Grad-CAM. It enhances the interpretability of the model, ensuring that the AGS network bases its decisions on relevant medical features, which increases its reliability for real-world clinical applications.

## 5. Results

In this paper, we evaluated the performance of eight classifiers using standard metrics commonly applied in deep learning. We demonstrate the classification performance of skin lesions by calculating Accuracy, Recall, Precision, and F1-Score using True Positives (TPs), False Negatives (FNs), False Positives (FPs), and True Negatives (TNs). Furthermore, to extend our evaluation to multi-class classification, we used the weighted average approach. This ensures that performance metrics like Precision, Recall, and F1-Score reflect the overall model performance, rather than being disproportionately influenced by the majority class.

### 5.1. Overall Performance

Table 4 presents the overall performance of eight classifiers—GoogLeNet, DenseNet201, ResNet50, MobileNet V3, EfficientNet B0, ViT, Swin Transformer, and EfficientNet V2—when tested with the AGS network for skin lesion classification.

The performance of each configuration is reported in terms of four key metrics: Accuracy, Precision, Recall, and F1-Score. In the table, the bold text highlights the best-performing model across all classifiers. The Gain values presented in the table quantify the improvements that the AGS network brings compared to the baseline models. These are the differences in the performances between the model in the first line, where no modules are applied, and the model in the second line, where the AGS network is applied. This highlights the effectiveness of the proposed module combination for enhancing classification performance.

Across the classifiers, a clear trend emerges: the inclusion of the AGS network consistently improves performance across all four metrics. The most significant improvement is observed with EfficientNet V2, where the EfficientNet V2 + AGS configuration outperforms the baseline model, achieving gains of +0.1808 in Accuracy, +0.1121 in Precision, +0.1808 in Recall, and +0.1674 in F1-Score. This result highlights the substantial advantage of incorporating the AGS network.

In terms of overall performance, ResNet50 stands out as the most appropriate classifier for the AGS network. It achieves the best overall performance across all metrics, as indicated in bold values for Accuracy (0.9587), Precision (0.9581), Recall (0.9587), and F1-Score (0.9573). These results suggest that ResNet50, when combined with the AGS network, provides the most optimal performance for skin lesion classification. Compared to the baseline model, this represents increases of 4.51% in Accuracy, 5.07% in Precision, 4.51% in Recall, and 4.78% in F1-Score. Note that we used the weighted average approach in this research. According to the official Python documentation (https://scikit-learn.org/stable/modules/generated/sklearn.metrics.recall_score.html, accessed on 19 December 2024) in multi-class classification problems, particularly when there is no significant imbalance between precision and recall (i.e., when the classifier is not heavily biased towards false positives or false negatives), the weighted recall often becomes equivalent to accuracy. As a result, our recall values in Table 4 are the same as the accuracy values.

Following ResNet50, ViT also demonstrated strong performance, with only a slight difference across the metrics. Under the AGS combination, ViT achieved Accuracy (0.9546), Precision (0.9561), Recall (0.9546), and F1-Score (0.9538). Additionally, EfficientNet V2 also showed outstanding results, achieving Accuracy (0.9510), Precision (0.9497), Recall (0.9510), and F1-Score (0.9498). These results show that while ResNet50 exhibited the best overall performance, ViT and EfficientNet V2 were close contenders, further validating the effectiveness of the AGS network.

To validate the performance improvements achieved by the AGS network, paired *t*-tests were conducted to compare baseline models with AGS-applied models across four metrics: Accuracy, Precision, Recall, and F1-Score. The results, summarized in Table 5, indicate that all *p*-values were less than 0.05, statistically significant improvements. These findings confirm that the AGS network consistently enhances model performance, further supporting its effectiveness in skin lesion classification. In addition, prior to conducting the paired *t*-tests, the normality of the differences in the data was verified using the Shapiro–Wilk test. The results for Accuracy, Precision, Recall, and F1-Score were 0.1440, 0.0964, 0.1440, and 0.1434, respectively, all exceeding the 0.05 threshold and confirming that the normality assumption was satisfied.

### 5.2. Training and Validation Loss Analysis

**Figure 6:**
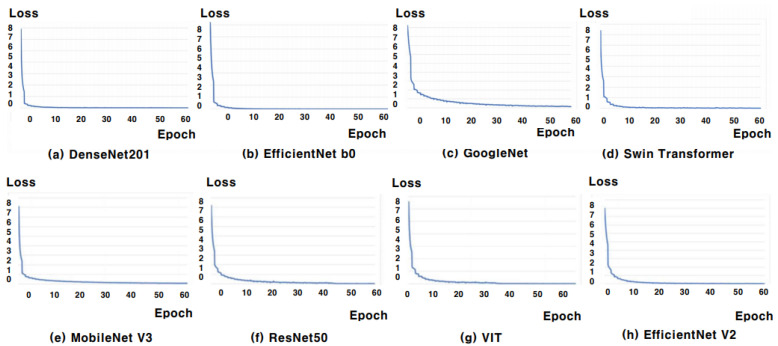
Training loss curves for eight deep learning models across 50 epochs. The models include (**a**) DenseNet201, (**b**) EfficientNet b0, (**c**) GoogleNet, (**d**) MobileNet v3, (**e**) ResNet50, (**f**) VIT, (**g**) Swin Transformer, and (**h**) EfficientNet V2. The x-axis represents the number of epochs, and the y-axis represents the training loss. These curves demonstrate that the models have successfully minimized the training loss, converging steadily as the number of epochs increases.

**Figure 7:**
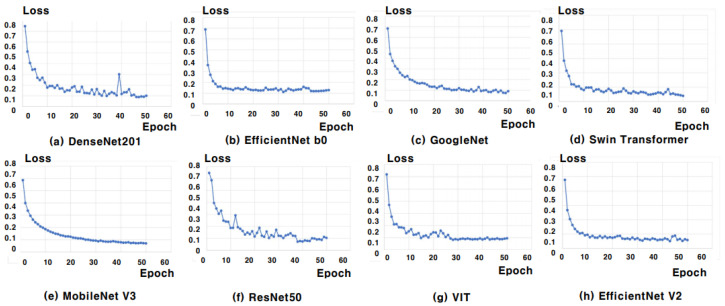
Validation loss curves for eight deep learning models across 50 epochs. The models include (**a**) DenseNet201, (**b**) EfficientNet b0, (**c**) GoogleNet, (**d**) MobileNet v3, (**e**) ResNet50, (**f**) VIT, (**g**) Swin Transformer, and (**h**) EfficientNet V2. The x-axis represents the number of epochs, and the y-axis represents the validation loss. These curves illustrate the models’ ability to generalize to unseen validation data, with each model showing distinct convergence behavior.

Figure 6 and Figure 7 show the training and validation loss curves for eight different classifiers—DenseNet201, EfficientNet B0, GoogLeNet, MobileNet V3, ResNet50, VIT, Swin Transformer, and EfficientNet V2—trained with the AGS network. Figure 6 presents the training loss curves, where all classifiers show a rapid drop in loss during the early epochs, followed by stabilization, confirming that the models quickly and effectively learn from the training data. In Figure 7, the validation loss consistently decreased for all eight classifiers, demonstrating well-converged validation loss curves. This indicates that each model is effectively learning from the data and improving its generalization to unseen validation data. The steady reduction in validation loss further suggests that the models are not overfitting, instead achieving better performance as training progresses. The overall alignment between training and validation losses suggests that the AGS network promotes efficient learning across all classifiers, with solid generalization performance for skin lesion classification.

### 5.3. Ablation Study on Impact of Module Combinations

We conducted additional experiments to analyze the influence of each module within the AGS network. Table 6 provides a comprehensive comparison of eight classifiers—GoogLeNet, DenseNet201, ResNet50, MobileNet V3, EfficientNet B0, ViT, Swin Transformer, and EfficientNet V2—tested with various combinations of three modules (A, G, S) for skin lesion classification. Each classifier was evaluated in five different configurations: (1) the base model, (2) the model with Module A, (3) the model with both Modules A and G, (4) the model with both Modules A and S, and (5) the model with Modules A, G, and S, which is our proposed AGS network.

The performance of each configuration is reported in terms of four key metrics: Accuracy, Precision, Recall, and F1-Score. In the table, the bold text highlights the best-performing configuration for each classifier, while the underlined values represent the highest overall performance across all classifiers.

The results in Table 6 demonstrate that applying Module A alone improves performance across all classifiers, compared to the baseline model. Therefore, it would be a reasonable choice to apply Module A to any classifier.

Next, we applied additional Modules G and S, denoted as AG and AS, respectively, in Table 6. However, the results show that Module AG or AS does not always guarantee improved performance. For instance, in the case of MobileNet v3, the Accuracy and F1 scores for Module AG are 0.9085 and 0.9023, respectively, and for Module AS, the values are 0.9034 and 0.9000. These results are lower compared to the values achieved with only Module A applied, where Accuracy and F1 scores are 0.9118 and 0.9089, respectively. In particular, Module AG shows a more noticeable tendency for performance degradation. Not only in MobileNet v3 but also in DenseNet201 and ViT, performance reduction is observed when AG is applied, compared to when only Module A applied. Finally, the AGS network, where all three modules A, G, and S are applied together, achieved the best classification performance across all eight classifiers.

In summary, despite often performance reductions with Module AG and Module AS, the AGS network consistently demonstrates the best performance across all classifiers. This indicates that the three modules exhibit optimal synergy when used together, and integrating all three modules is significant for achieving superior performance. Notably, the AGS network outperforms any other combination across all eight classifiers, confirming its effectiveness in enhancing skin lesion classification.

### 5.4. Grad-CAM Analysis

To verify whether the best-performing ResNet50 with AGS network model accurately recognizes and classifies skin lesions, we utilized Grad-CAM (Gradient-weighted Class Activation Mapping) to visualize the model’s focus during the classification process. Grad-CAM helps generate a heatmap of the areas in the image that the model considered most important for making its prediction.

Grad-CAM computes gradients for the feature maps of the final convolutional layer in the ResNet50 architecture. By using the variation values (gradients) of the last layer, Grad-CAM captures the spatial regions that contribute the most to the predicted class score. In this research, the heatmap highlights the most discriminative features of the lesions, allowing us to visually inspect whether the model’s decision-making process aligns with human intuition and medical understanding.

**Figure 8:**
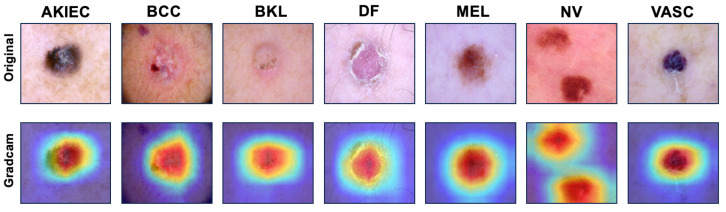
Grad-CAM visualizations of skin lesion classifications for seven skin cancers using ResNet50 with the AGS network.

As shown in Figure 8, the heatmaps generated by Grad-CAM focus on the core areas of each lesion. It indicates that the ResNet50 + AGS network correctly identifies the relevant features of the skin lesions. By visualizing where the model concentrates its attention, we can ensure that the ResNet50 with AGS network is not relying on irrelevant background information but is instead correctly focusing on the lesion areas that a human expert would consider significant for diagnosis.

## 6. Discussion

In this study, we addressed the limitations of previous research, which primarily focused on single augmentation methods, by introducing the AGS network, an integrated approach that combines multiple augmentation techniques to enhance the performance of skin lesion classifiers. Additionally, we analyzed the individual performance contributions of each module within the AGS network. The results clearly demonstrate the effectiveness of the AGS network in improving skin lesion classification, but there are some limitations and areas for further exploration.

First, utilizing larger and more diverse datasets will be crucial to confirm the robustness and generalizability of the AGS network. However, when data are collected using different types of microscopes or under varying clinical environments, appropriate preprocessing steps will be essential to ensure the model’s consistent and efficient application.

Second, further experiments on hyperparameter tuning and its impact on model performance are necessary. It is important to explore the optimal parameters that can deliver the best performance across various classifiers. Additionally, it will be essential to determine whether the proposed model remains robust to changes in hyperparameters, ensuring consistent and reliable performance under different configurations.

Another important aspect is model interpretability, which remains crucial for applying AI in healthcare. While Grad-CAM provided valuable insights into the model’s decision-making process, incorporating more advanced interpretability techniques, such as DeepLIFT and Score-CAM, could further enhance our understanding of deep learning models in clinical settings. Ensuring transparency and reliability in these models is key to gaining trust in AI-driven diagnostic tools.

In addition, each module requires further in-depth exploration and refinement of its underlying algorithms. For Module A (Augmentation), the current approach incorporates direction and color changes in the images, reflecting clinical scenarios where a doctor might view the same lesion from multiple directions or where a similar lesion may appear in a flipped orientation. Additionally, when using a dermoscope, variations in lens quality can cause noticeable color differences. To create a more robust model capable of handling such diverse environments, basic augmentations were randomly applied. For Module S (Segmentation), U-Net was selected after evaluating its performance against other models, as shown in Table 2, to prevent the model from focusing on the periphery rather than the lesion itself. However, the correlation or justification between the selection of modules A and G and clinical necessity remains somewhat weak, requiring further validation and clinical persuasiveness. For instance, in clinical practice, when diagnosing malignant melanoma, symmetry or the outline shape of a lesion is often a critical factor. To better reflect such clinical considerations, integrating additional information, such as lesion border details, or incorporating algorithms that quantify symmetry, could be valuable for improving diagnostic performance. Considering such approaches would strengthen the clinical applicability of the AGS network.

Also, for Module G (GAN), PGGAN was adopted as the generative algorithm. However, additional experiments are required to explore other neural augmentation models including style GAN and diffusion models. Comparative analyses of various methods should be conducted to verify their efficacy and to identify the most suitable generative approach for clinical datasets.

Finally, for the practical implementation of this methodology, it is essential to develop user-friendly software that can be easily used by healthcare professionals and even individuals without expertise in computer science or image processing. While model training may require expert involvement, the inference process could be simplified so that users can upload an image and receive an analysis of the lesion. We believe this approach could be particularly valuable in skin lesion cases, where individuals might neglect symptoms and avoid visiting a doctor. As an initial self-diagnosis tool, the AGS network has the potential to provide significant value in both clinical and non-clinical settings.

## 7. Conclusions

In this paper, we suggested a comprehensive approach, the AGS network, which integrates Augmentation (Module A), GAN (Module G), and Segmentation (Module S) for enhancing the skin cancer dataset. We explored the effectiveness of our AGS network in improving skin lesion classification by testing various combinations of modules across eight deep learning classifiers: GoogLeNet, DenseNet201, ResNet50, MobileNet V3, EfficientNet B0, ViT, Swin Transformer, and EfficientNet V2. Our results consistently demonstrated that the AGS network significantly enhances the performance of these classifiers in terms of Accuracy, Precision, Recall, and F1-Score. Notably, ResNet50 with the AGS network combination emerged as the top-performing model, achieving the highest values across all metrics, indicating its suitability for this task.

We also validated the contribution of each module—A, G, and S—to the overall performance. While the application of Module A alone consistently improved performance over the base models, the combinations of AG and AS did not always guarantee performance gains, underscoring the necessity of using all three modules together. The AGS combination brought about significant improvements, as shown by the Gain values, which quantify the differences between the baseline models and the AGS-enhanced model. These results show that the combined effect of all three modules is greater than their individual contributions, leading to optimal classification performance. We hope that the AGS network will contribute meaningfully to the field of medical image classification, especially for tasks such as skin lesion diagnosis. Furthermore, future research could build upon this work by applying the AGS framework to other medical imaging tasks, expanding the versatility and impact of this approach in clinical settings.

## Figures and Tables

**Table 1:** Summary of previous research on skin lesion classification using the HAM10000 dataset.

Author	Model/Method	Dataset	Accuracy (%)
Rahman et al. [21]	Weighted Ensemble Technique	HAM10000	85.8%
Emrah et al. [22]	Pre-trained VGGNET-16 Architecture	HAM10000	85.62%
Chaturvedi et al. [23]	Transfer Learning	HAM10000	83.1%
Hassan et al. [25]	Densely Connected Convolutional Network	HAM10000	92%
Anand et al. [31]	Modified MobileNet Architecture	HAM10000	90%
Khan et al. [32]	Using Deep Learning Features and Improved Moth Flame Optimization	HAM10000	90.67%
Khan, Zhang and sharif et al. [16]	24-layered Convolutional Neural Network Architecture	HAM10000	86.5%
Gangwani et al. [34]	Transfer Learning and Data Augmentation	HAM10000	91%
Syed et al. [34]	Densely Connected Convolution Network	HAM10000	92%

**Table 2:** Performance comparison of segmentation models (U-Net, Mask R-CNN, and DeepLab) based on Intersection-over-Union (IoU) and Dice coefficient metrics. All models were trained for 10 epochs using the Adam optimizer with a learning rate of $\left(1\times 10^{-4}\right)$. The best-performing values are highlighted in bold.

Model	Intersection-over-Union (IoU)	Dice Coefficient
**U-Net**	**0.8964**	**0.9406**
Mask R-CNN	0.8647	0.9198
DeepLab	0.8594	0.9218

**Table 3:** Summary of model training parameters and settings.

Parameter	Value
Image Size	(3 × 224 × 224)
Train/Validation/Test (Ratio)	8:1:1
Batch Size	32
Optimizer	Adam
Learning Rate	1 $\times \;10^{-5}$
Cross-Validation	5-Fold
Loss Function	CrossEntropyLoss
Epochs	50
Transfer Learning	“YES”
Scheduler	ReduceLROnPlateau (mode = `min’, factor = 0.1 patience = 10)
Dataset	HAM10000 dataset

**Table 4:** Overall performance of eight classifiers with the AGS network for skin lesion classification. The bold text highlights the best-performing model across all classifiers. The (weighted) recall value is equivalent to accuracy since we used the weighted average approach. The gain is calculated as the difference in performance between the baseline model and the model with the AGS network applied.

Classifier	Accuracy	Precision	Recall	F1
GoogleNet	0.9016	0.9001	0.9016	0.8988
GoogleNet + AGS (ours)	0.9296	0.9397	0.9296	0.9321
Gain	+0.0280	+0.0396	+0.0280	+0.0333
DenseNet201	0.8205	0.8019	0.8205	0.8034
DenseNet201 + AGS (ours)	0.9492	0.9492	0.9492	0.9479
Gain	+0.1287	+0.1473	+0.1287	+0.1445
ResNet50	0.9136	0.9074	0.9136	0.9095
**ResNet50 + AGS (ours)**	**0.9587**	**0.9581**	**0.9587**	**0.9573**
Gain	+0.0451	+0.0507	+0.0451	+0.0478
MobileNet v3	0.8584	0.8454	0.8584	0.8472
MobileNet v3 + AGS (ours)	0.9274	0.9278	0.9274	0.9252
Gain	+0.0690	+0.0824	+0.0690	+0.0780
EfficientNet b0	0.8853	0.8906	0.8853	0.8854
EfficientNet b0 + AGS (ours)	0.9299	0.9327	0.9299	0.9290
Gain	+0.0446	+0.0421	+0.0446	+0.0436
VIT	0.9220	0.9225	0.9220	0.9211
VIT + AGS (ours)	0.9546	0.9561	0.9546	0.9538
Gain	+0.0326	+0.0336	+0.0326	+0.0478
Swin Transformer	0.8076	0.8713	0.8076	0.8124
Swin Transformer + AGS (ours)	0.9310	0.9262	0.9310	0.9269
Gain	+0.1234	+0.0549	+0.1234	+0.1145
EfficientNet v2	0.7702	0.8376	0.7702	0.7824
EfficientNet v2 + AGS (ours)	0.9510	0.9497	0.9510	0.9498
Gain	+0.1808	+0.1121	+0.1808	+0.1674

**Table 5:** Results of paired *t*-test for the performance differences between the AGS-applied models and baseline models across eight algorithms.

Metric	t-Statistic	*p*-Value
Accuracy	−4.1187	0.0045
Precision	−4.9022	0.0017
Recall	−4.1187	0.0045
F1-Score	−4.4091	0.0031

**Table 6:** Performance comparison of various module combinations on eight classifiers for skin lesion classification. The bold text highlights the best-performing configuration for each classifier, while the underlined values represent the highest overall performance across all classifiers. The (weighted) recall value is equivalent to accuracy since we used the weighted average approach.

Classifier	Module A	Module G	Module S	Accuracy	Precision	Recall	F1
GoogleNet	-	-	-	0.9016	0.9001	0.9016	0.8988
GoogleNet + mod.A	✓	-	-	0.9140	0.9150	0.9140	0.9122
GoogleNet + mod.AG	✓	✓	-	0.9140	0.9195	0.9140	0.9143
GoogleNet + mod.AS	✓	-	✓	0.9154	0.9147	0.9154	0.9137
**GoogleNet + mod.AGS (ours)**	✓	✓	✓	**0.9296**	**0.9397**	**0.9296**	**0.9321**
DenseNet201	-	-	-	0.8205	0.8019	0.8205	0.8034
DenseNet201 + mod.A	✓	-	-	0.9205	0.9239	0.9205	0.9204
DenseNet201 + mod.AG	✓	✓	-	0.9162	0.9167	0.9162	0.9147
DenseNet201 + mod.AS	✓	-	✓	0.9365	0.9380	0.9365	0.9360
**DenseNet201 + mod.AGS (ours)**	✓	✓	✓	**0.9492**	**0.9492**	**0.9492**	**0.9479**
ResNet50	-	-	-	0.9136	0.9074	0.9136	0.9095
ResNet50 + mod.A	✓	-	-	0.9187	0.9208	0.9187	0.9153
ResNet50 + mod.AG	✓	✓	-	0.9191	0.9200	0.9191	0.9175
ResNet50 + mod.AS	✓	-	✓	0.9539	0.9538	0.9539	0.9523
**ResNet50 + mod.AGS (ours)**	✓	✓	✓	**0.9587**	**0.9581**	**0.9587**	**0.9573**
MobileNet v3	-	-	-	0.8584	0.8454	0.8584	0.8472
MobileNet v3 + mod.A	✓	-	-	0.9118	0.9114	0.9118	0.9089
MobileNet v3 + mod.AG	✓	✓	-	0.9085	0.9015	0.9085	0.9023
MobileNet v3 + mod.AS	✓	-	✓	0.9034	0.9043	0.9034	0.9000
**MobileNet v3 + mod.AGS (ours)**	✓	✓	✓	**0.9274**	**0.9278**	**0.9274**	**0.9252**
EfficientNet b0	-	-	-	0.8853	0.8906	0.8853	0.8854
EfficientNet b0 + mod.A	✓	-	-	0.9132	0.9176	0.9132	0.9110
EfficientNet b0 + mod.AG	✓	✓	-	0.9169	0.9152	0.9169	0.9133
EfficientNet b0 + mod.AS	✓	-	✓	0.9216	0.9189	0.9216	0.9181
**EfficientNet b0 + mod.AGS (ours)**	✓	✓	✓	**0.9299**	**0.9327**	**0.9299**	**0.9290**
VIT	-	-	-	0.9220	0.9225	0.9220	0.9211
VIT + mod.A	✓	-	-	0.9227	0.9289	0.9227	0.9234
VIT + mod.AG	✓	✓	-	0.9227	0.9220	0.9227	0.9208
VIT + mod.AS	✓	-	✓	0.9525	0.9529	0.9525	0.9514
**VIT + mod.AGS (ours)**	✓	✓	✓	**0.9546**	**0.9561**	**0.9546**	**0.9538**
Swin Transformer	-	-	-	0.8076	0.8713	0.8076	0.8124
Swin Transformer + mod.A	✓	-	-	0.8174	0.8926	0.8174	0.8347
Swin Transformer + mod.AG	✓	✓	-	0.8984	0.9035	0.8984	0.8953
Swin Transformer + mod.AS	✓	-	✓	0.9220	0.9156	0.9220	0.9157
**Swin Transformer + mod.AGS (ours)**	✓	✓	✓	**0.9310**	**0.9262**	**0.9310**	**0.9269**
EfficientNet v2	-	-	-	0.7702	0.8376	0.7702	0.7824
EfficientNet v2 + mod.A	✓	-	-	0.7866	0.8599	0.7866	0.8012
EfficientNet v2 + mod.AG	✓	✓	-	0.8207	0.8848	0.8207	0.8394
EfficientNet v2 + mod.AS	✓	-	✓	0.9296	0.9268	0.9296	0.9259
**EfficientNet v2 + mod.AGS (ours)**	✓	✓	✓	**0.9510**	**0.9497**	**0.9510**	**0.9498**

## Data Availability

The HAM10000 dataset used in this study is publicly available and can be downloaded from the following link: https://dataverse.harvard.edu/dataset.xhtml?persistentId=doi:10.7910/DVN/DBW86T (accessed on 19 December 2024).
